# Paracoccidioidomycosis after Highway Construction, Rio de Janeiro, Brazil

**DOI:** 10.3201/eid2311.170934

**Published:** 2017-11

**Authors:** Antonio C. Francesconi do Valle, Priscila Marques de Macedo, Rodrigo Almeida-Paes, Anselmo R. Romão, Marcia dos Santos Lazéra, Bodo Wanke

**Affiliations:** Evandro Chagas National Institute of Infectious Diseases, Rio de Janeiro, Brazil (A.C. Francesconi do Valle, P. Marques de Macedo, R. Almeida-Paes, M. dos Santos Lazéra, B. Wanke);; Institute of Scientific and Technological Communication and Information in Health, Rio de Janeiro (A.R. Romão)

**Keywords:** paracoccidioidomycosis, Paracoccidioides, fungi, conidia, systemic mycosis, neglected disease, human infections, respiratory infections, outbreak, soil, aerial dispersion, highway construction, deforestation, Rio de Janeiro, Brazil

## Abstract

Transmission of *Paracoccidioides* spp. fungi to humans is usually related to manipulation of soil. Rural workers are the most affected group. We report an outbreak of paracoccidioidomycosis after deforestation and massive earth removal during construction of a highway in Rio de Janeiro, Brazil. Extensive environmental disturbances might be involved in fungal transmission.

Paracoccidioidomycosis is the major systemic mycosis in Latin America and the leading fungal cause of death in immunocompetent persons in Brazil (*1**,**2*). Paracoccidioidomycosis is a neglected disease whose prevalence and incidence rates are underestimated because of lack of mandatory reporting. Infection follows inhalation of *Paracoccidioides* spp. conidia in the soil (*3**,**4*) and can progress to disease, typically manifested in 1 of 2 clinical forms. The first form is chronic (adult type), which accounts for ≈80% of paracoccidioidomycosis cases, mostly in rural workers who show fungal endogenous reactivation in the lungs and other organs later in life. The second form is acute/subacute (juvenile type), which occurs primarily in young patients and is more severe because of progressive reticuloendothelial involvement, which results in high rates of complications, including death (*5*).

There have been reports of *Paracoccidioides* spp. infections after disturbances of soil that resulted in aerial dispersion of fungal propagules. Native indigenous populations in Latin America changed their ancient livelihood practices to cultivate coffee after deforestation of the Amazon rainforest, which resulted in paracoccidioidomycosis infections (*6**,**7*). In addition, climate changes related to the El Niño events, such as a high rainfall index followed by increased storage of water by soil and higher humidity, have been shown to occur before an increase of acute/subacute paracoccidioidomycosis cases (*8*).

We report an outbreak of paracoccidioidomycosis after deforestation and massive earth removal during construction of a highway in Rio de Janeiro, Brazil. The study protocol was approved by the Evandro Chagas National Institute of Infectious Diseases Research Ethics Committee (register CAAE 42590515.0.0000.5262).

The Evandro Chagas National Institute of Infectious Diseases in Rio de Janeiro is a reference center for paracoccidioidomycosis. This disease is endemic to the state of Rio de Janeiro (*5*). During 1988–2015, the annual average number of acute/subacute cases of paracoccidioidomycosis at this institution was 2.3 cases/year for this state and 1.4 cases/year for Baixada Fluminense, a region composed of 12 municipalities in the metropolitan area of Rio de Janeiro (Figure). However, during December 2015–December 2016, a total of 8 cases were diagnosed at this center, all from Baixada Fluminense, a rate ≈5.7 times higher than that expected for this period. The most recent (2016) census in Brazil estimated that there were 968,680 persons <30 years of age living in the affected municipalities (*9*).

**Figure:**
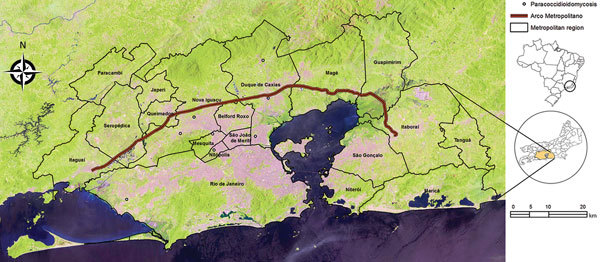
Metropolitan area of Rio de Janeiro, Brazil, showing the Raphael de Almeida Magalhães Highway, also known as the Arco Metropolitano, and georeferenced cases of paracoccidioidomycosis (open circles) during highway construction. Inset shows location of metropolitan area in Rio de Janeiro State and of Rio de Janeiro State (circle) in Brazil. Source: Landsat 8 Images (https://earthexplorer.usgs.gov/).

Case definition was based on clinical and laboratory criteria: reticuloendothelial involvement in young patients; laboratory test results confirming the presence of multibudding yeast-like *Paracoccidioides* cells by direct microscopy or histopathologic analysis; fungal isolation in culture; or a positive serologic result for paracoccidioidomycosis (*3*). Data for the 8 case-patients (4 males and 4 females) are provided (Technical Appendix Table 1). Mean age was 22 (range 10–28) years. Median time for diagnosis was 7 (range 4–16) months. Predominant clinical manifestations were cervical lymph node enlargement (100%), hepatomegaly (25%), and splenomegaly (25%). Serious complications occurred in 5 patients: adrenal insufficiency (3 patients), cholestasis (1 patient), esophageal fistula (1 patient), and acute upper airway obstruction (1 patient). A 19-year-old patient died because of complications of paracoccidioidomycosis.

The Raphael de Almeida Magalhães Highway, also known as Arco Metropolitano, is a new highway in the study region (Figure). During its construction (2008–2014), large areas were deforested and massive amounts of earth were removed, which resulted in discovery of 62 archeological sites through 2012 (*10*). Two thirds of this highway (97 km) was constructed during 2012–2014. The highway was complete for 1 year before the number of new cases of paracoccidioidomycosis increased. Residences of patients were 0.1 km–16.6 km from construction areas. The increase in the number of acute/subacute cases of paracoccidioidomycosis, with temporal and geographic relationships to this construction, suggests a possible new risk for outbreaks of paracoccidioidomycosis.

Other hypotheses for this cluster are clearing of forests, soil humidity, and the El Niño phenomenon (*3**,**8*). It is noteworthy that the highway crosses a native Atlantic forest area. Moreover, over several months in 2013, this region had high rainfall indexes (Technical Appendix Table 2), which presumably contributed to retention of moisture in the soil. A previous study showed that soil humidity favors sporulation and dispersal of *Paracoccidioides* spp. (*3*). Also, a high-intensity El Niño phenomenon occurred during May 2015–March 2016.

The incidence of acute paracoccidioidomycosis in the affected area after highway construction (8.25 cases/1 million persons/y, 95% CI 4.18–16.3 cases/1 million persons/y) was higher than that before highway construction (1.29 cases/1 million persons/y, 95% CI 0.74–4.03 cases/1 million persons/y). More persons were probably exposed to *Paracoccidioides* conidia, but these persons did not show progression/development of disease, did not seek medical attention, and did not have cases reported to health authorities. The chronic form of paracoccidioidomycosis will probably develop in some of these patients.

This study underscores the need for paracoccidioidomycosis surveillance, especially in the context of environmental alterations enhanced by climate change and affected by construction, deforestation, and other human interventions. Enhanced surveillance will more fully identify relative risks of different human enterprises and facilitate interventions for at-risk populations to reduce and prevent future outbreaks of paracoccidioidomycosis.

Technical Appendix Additional information on paracoccidioidomycosis after highway construction, Rio de Janeiro, Brazil.
