# Supplementary material for: Paracoccidioidomycosis after Highway Construction, Rio de Janeiro, Brazil
Source: Emerg Infect Dis. 2017 Nov;23(11):1917–9. doi: 10.3201/eid2311.170934 (PMC5652422; doi:10.3201/eid2311.170934)
Supplement: Technical Appendix — Additional information on paracoccidioidomycosis after highway construction, Rio de Janeiro, Brazil. [file 17-0934-Techapp-s1.pdf]

Article DOI: https://doi.org/10.3201/eid2311.170934

Paracoccidioidomycosis during Highway Construction, Rio de Janeiro, Brazil

Technical Appendix.

Technical Appendix Table 1. Characteristics for 8 patients with paracoccidioidomycosis during highway construction, Rio de Janeiro, Brazil*

Patient no.	Age, y/sex	Residence distance from highway, km	Municipality/year of highway construction	Symptom onset/exposure time, mo	Date of diagnosis	Diagnostic technique/sample	Complication /outcome
1	31/F	9.8	DC/2013	2015 Apr/≤27	2015 Dec	Culture/lymph node aspirate	None/clinical cure, still being treated
2	21/F	9.5	NI/2013	2014 Aug/≤19	2016 Jan	Histologic analysis/lymph node for biopsy	Adrenal insufficiency/ clinical cure, still being treated
3	28/F	9.3	MES/2013	2015 Jul/≤30	2016 Feb	Histologic analysis/lymph node and spleen sample for biopsy	Adrenal and respiratory insufficiency, tracheostomy/ clinical improvement, treatment suspended (pregnancy)
4	27/M	4.7	NI/2013	2015 Dec/≤35	2016 Mar	Culture/lymph node aspirate	None/clinical cure, still being treated
5	23/F	0.1	DC/2013	2015 Apr/≤27	2016 Apr	Direct examination/skin sample for biopsy	Adrenal insufficiency/ clinical cure, still being treated
6	23/M	12.3	DC/2013	2015 Dec/≤35	2016 Jun	Culture/lymph node aspirate	Cholestasis/ clinical cure, still being treated
7	19/M	14.2	NI/2013	2016 Jan/≤36	2016 Jun	Culture/lymph node aspirate and blood	Vasculitis, esophageal fistulae/died
8	10/M	16.6	SEP†/2013	2016 Jul/≤42	2016 Oct	Serologic analysis/blood	NA (lost to followup)

*Exposure to *Paracoccidioides* conidia might may have occurred during highway construction or subsequently as a consequence of massive earth removal from areas with native forests where the fungus was present. This anthropic activity might result in a recurrent or continuous aerial dispersion of fungal propagules during and after construction. The incubation time for acute/subacute paracoccidioidomycosis is unknown, but it might be long because there are no reports of this disease in children <2.5 years of age, in contrast with other pulmonary systemic mycoses, such as histoplasmosis, coccidioidomycosis, and blastomycosis. DC, Duque de Caxias; MES, Mesquita; NA, not available; NI, Nova Iguaçu; NIL, Nilópolis; SEP, Sepetiba.
†Although the residence of patient 8 was in Sepetiba, he had also lived in Duque de Caxias.

Technical Appendix Table 2. Observed and expected monthly rainfall indexes for Duque de Caxias, Rio de Janeiro, Brazil, 2012–2016*

Year	Month, mm												Total, mm
	Jan	Feb	Mar	Apr	May	Jun	Jul	Aug	Sep	Oct	Nov	Dec	
2012	NA	NA	NA	NA	141.2	143.6	45.8	33.6	140.4	95.8	241.2	129.2	>970.8
2013	590.2	91.2	471.2	127.0	158.2	66.2	188.6	39.8	87.8	102.6	260.0	318.2	2,501.0
2014	67.4	50.8	160.2	215.0	75.4	99.8	50.2	58.8	51.0	110.6	196.2	105.2	1,240.6
2015	141.8	0	229.4	70.2	**43.0**	**157.0**	**34.2**	**21.0**	**138.1**	**52.8**	**251.8**	**111.4**	1,250.7
2016	**528.6**	**344.8**	**199.0**	12.6	65.6	161.8	8.0	68.8	123.8	116.2	650.0	244.0	2,523.2
Expected	366.3	176.2	270.9	183.7	111.0	80.6	85.4	64.2	110.2	101.4	341.5	330.5	2,221.9

*Measurements in 2012 started in May. Bold indicates months when the El Niño phenomenon occurred. NA, not available.
